# ‘I hated being ghosted’ – The relevance of social participation for living well with post‐stroke aphasia: Qualitative interviews with working aged adults

**DOI:** 10.1111/hex.13291

**Published:** 2021-06-15

**Authors:** Molly Manning, Anne MacFarlane, Anne Hickey, Rose Galvin, Sue Franklin

**Affiliations:** ^1^ School of Allied Health Faculty of Education and Health Sciences & Health Research Institute University of Limerick Limerick Ireland; ^2^ School of Medicine Faculty of Education and Health Sciences & Health Research Institute University of Limerick Limerick Ireland; ^3^ Department of Psychology Royal College of Surgeons in Ireland Dublin Ireland

**Keywords:** Aphasia, patient involvement, rehabilitation, social participation, social support, stroke

## Abstract

**Background:**

In the context of increasing incidence of stroke in working aged adults, there is a specific need to explore the views of working aged adults with post‐stroke aphasia, whose communication difficulties may result in restricted social participation, loss of employment and changed relationship and parenting roles. This study aimed to explore the perspectives of working aged adults with post‐stroke aphasia in relation to social participation and living well with aphasia (LWA).

**Design and participants:**

We conducted qualitative interviews with 14 people with post‐stroke aphasia (PWA). Data were analysed following principles of reflexive, thematic analysis.

Patient or Public Contribution: A Public and Patient Involvement aphasia advisory group inputted into the study design and interpretation of data.

**Results:**

Social participation spanned 5 themes: Relationships and roles; Social support; Peer network, Aphasia awareness; and Employment and training. Meaningful, interesting social participation for LWA is individually defined. Working aged PWA may require flexible support with parenting, accessing a diverse social network and finding opportunities for meaningful social connection, training and employment.

**Conclusions:**

The findings extend knowledge of social participation in the context of LWA for working aged adults by elucidating the individually defined nature of meaningful participation and how PWA may need flexible support with parenting, accessing a diverse social network and training and employment. For aphasia research, policy and services to be relevant, it is crucial that working aged PWA are meaningfully involved in setting the aphasia agenda.

## 1 INTRODUCTION

Aphasia is an acquired language disorder, most commonly occurring post‐stroke, that ‘masks a person's inherent competence, and most dramatically affects conversational interaction (talking and understanding), as well as the ability to read and write’.^1^, ^2^ Aphasia has far‐reaching psychosocial consequences for quality of life, mental health, social networks, relationships, return to work, access to support and social participation.^3^, ^4^, ^5^, ^6^, ^7^, ^8^, ^9^, ^10^, ^11^, ^12^, ^13^, ^14^, ^15^, ^16^ There is a growing research focus on supporting people with post‐stroke aphasia (PWA) to achieve participation outcomes in addition to impairment‐focused rehabilitation approaches.^17^, ^18^ Such interventions may include raising aphasia awareness, improving communicative accessibility, targeting social inclusion, communication partner training and connecting PWA with opportunities for authentic, meaningful social participation.^19^, ^20^, ^21^, ^22^, ^23^

A recent qualitative evidence synthesis of 31 articles reporting the perspectives of 350+ PWA highlighted the importance of social participation for living well with aphasia (LWA).^24^ The findings were limited in that they were drawn from studies examining a diverse range of topics in addition to LWA. Shortcomings in the literature were also highlighted including a lack of public and patient involvement of PWA in aphasia research, despite empirical evidence of successful participatory research in the context of aphasia.^25^, ^26^, ^27^, ^28^

A little under half of participants (42%) in the included papers were clearly documented as working age. Aphasia brings unique, inter‐connected consequences for social participation for working aged adults who will live with chronic aphasia for a longer number of years^29^, ^30^ and so it is particularly important to increase understanding of supporting this age group. This is especially salient in the context of the growing incidence of stroke in working aged people^31^, ^32^, ^33^ and evidence that access to stroke support is front‐loaded.^34^, ^35^, ^36^, ^37^, ^38^, ^39^, ^40^, ^41^, ^42^, ^43^

We therefore aimed to address relative under‐representation of the perspectives of working aged PWA, including those with severe aphasia, towards social participation and LWA. We designed a qualitative interview study with input from a Public and Patient Involvement (PPI) aphasia advisory group, which generated a rich data set about multiple aspects of LWA. This paper focuses on the relevance of social participation for LWA.

## 2 MATERIALS AND METHODS

### 2.1 Qualitative approach and research paradigm

The study design was qualitative, semi‐structured, in‐depth interviews.^44^ Our approach was framed by Critical Realism, as detailed previously.^45^ We have followed the Standards for Reporting Qualitative Research (SRQR)^46^ (Supplementary file 1).

### 2.2 Researcher characteristics and reflexivity

2.2

The multi‐disciplinary team included expertise in aphasia, PPI, health psychology and primary care and four PPI collaborators (3 women, 1 man) of working age living with post‐stroke aphasia for 3–30 years.

### 2.3 Ethical approval

We obtained approval from the Research Ethics Committees of the Faculty of Education & Health Sciences, University of Limerick (REC Ref: 2016_09_06_EHS), University Hospital (REC Ref: 124/16), Acquired Brain Injury Ireland (ABII) and Headway. We also obtained study approval from Aphasia Ireland, Croí, Irish Heart Foundation (IHF) and Limerick Stroke Club.

### 2.4 Sampling strategy

We circulated recruitment information (Supplementary file 2) to Speech and Language Therapists (SLTs) in the Health Service Executive and various third‐sector support organizations (ABII, Headway, Aphasia Ireland, Croí, IHF), who identified potential participants within their organization. The sampling parameters were working aged adults (18–65 years) with post‐stroke aphasia, a minimum of 1 year post‐onset. To focus on the experiences of PWA, we excluded people with severe cognitive or hearing impairment. We used maximal variation sampling to maximize diversity by age, sex, location, referring organization, severity of aphasia, years since stroke and living situation.^47^ All eligible and interested participants presenting within a 5‐month window in 2019 were recruited to the study (to enable the project to be completed within the allocated time available). However, participant diversity was actively monitored throughout.

At an initial meeting, the first author obtained informed consent by reading the information sheet and consent form aloud and ensuring that participants had comprehended each element. We also administered subtests of the Boston Diagnostic Aphasia Exam,^48^ the Western Aphasia Battery – Revised^49^ and the Comprehensive Aphasia Test^50^ to determine aphasia severity and communication support needs. Each PWA could invite a significant other who would attend the interview to help support their communication.^13^ This option was explained and explored with the participant with aphasia. Written informed consent was subsequently obtained from significant others at the time of interview.

### 2.5 Data collection

Interviews were conducted by the first author (trained SLT) either in participant homes or third‐sector support organizations. Interviews were conducted at least 2 days after initial meeting except for 3 participants, for whom fatigue was not an issue, who were interviewed at initial meeting. Interview length averaged 96 min (range 50–128). Interviews were audio and video recorded. The researcher monitored for signs of fatigue, emotional distress and breaks were taken whenever required.^51^, ^52^ The topic guide published previously^45^ was developed and piloted with PPI advisors in practice interviews. The topic guide facilitated semi‐structured interviews without fixed sequencing or wording and using non‐directed open questions where possible, but allowing for scaffolding where required, including binary choice alternatives and yes/no questions. Scaffolding also included providing examples of what other PWA had said in early interviews and in PPI meetings.^28^, ^53^ A description of the strategies we used to support PWA to participate in the interviews is published elsewhere.^45^ These included suggestions made by and piloted with our PPI contributors to ensure that significant others, when present, did not ‘speak for’ participants with aphasia.^52^ These included, for example, addressing questions in the first instance to the participant with aphasia by name, and verifying all significant other contributions with the participant with aphasia.

### 2.6 Participants

We interviewed 14 PWA (6 women, 8 men) and 4 spouses as a source of communication support. PWA was 33–62 years old and 14 months–14 years post‐stroke onset; six had severe aphasia including 3 with a severe receptive aphasia (Table 1). Eight participants had children at the time of stroke, including one who experienced stroke shortly after the birth of her baby and 4 single parents. Two more participants had adult children at the time of stroke.

**TABLE 1:** Participant characteristics

Maximum variation sampling variable		Number of participants (N = 14)
Gender	Male	8
	Female	6
Time since stroke	Mean (SD): 7 years (4 years); Range: 14 months–14 years	
	<2 years	2
	2–5 years	4
	6–10 years	5
	11+ years	3
Age	Mean (SD): 51 years old (8 years); Range: 33–62 years *[Age at stroke, Mean (SD):45 years old (10 years);* *Range: 23 ‐ 58 years]*.	
Aphasia severity	Mild	3
	Moderate	5
	Severe	6
Marital status	Single (unmarried)	4
	Separated / Divorced	4
	Married	6
Living situation	Living alone	2
	Lives with at least one other person	12
Referral source	Acquired Brain Injury Ireland (ABII)	2
	Aphasia Ireland	1
	Croí	3
	Headway	6
	HSE	1
	Irish Heart Foundation (IHF)	1
Currently employed	Yes	2
	No	12
Employed at stroke	Yes	13
	No	1

### 2.7 Data processing and analysis

The first author transcribed and imported data to NVivo11 for reflexive thematic analysis.^54^, ^55^, ^56^, ^57^ Transcripts were read in detail and initial observations and insights were noted. Initial coding involved a flexible deductive process, described elsewhere,^45^ applying 87 codes created in our earlier systematic review of living well with aphasia^24^ or inductively creating new ones. Preliminary themes were developed through axial coding and presented to the PPI group for further interpretation (Supplementary file 3). Data and coding within themes were analysed and inter‐relationships between themes were visualized. Themes were named and defined, and a written summary of the analysis grounded in participant data was constructed.^55^

### 2.8 Techniques to enhance trustworthiness

The first author met the second and final authors after coding 4 transcripts to discuss analytical method, and with the final author throughout analysis for in‐depth interrogation of the analytical insights. Together with PPI contributors, this collaborative approach helped provide ‘a richer more nuanced reading of the data’^56^ (p. 594). A detailed, reflexive audit trail was maintained.

## 3 RESULTS

**FIGURE 1:**
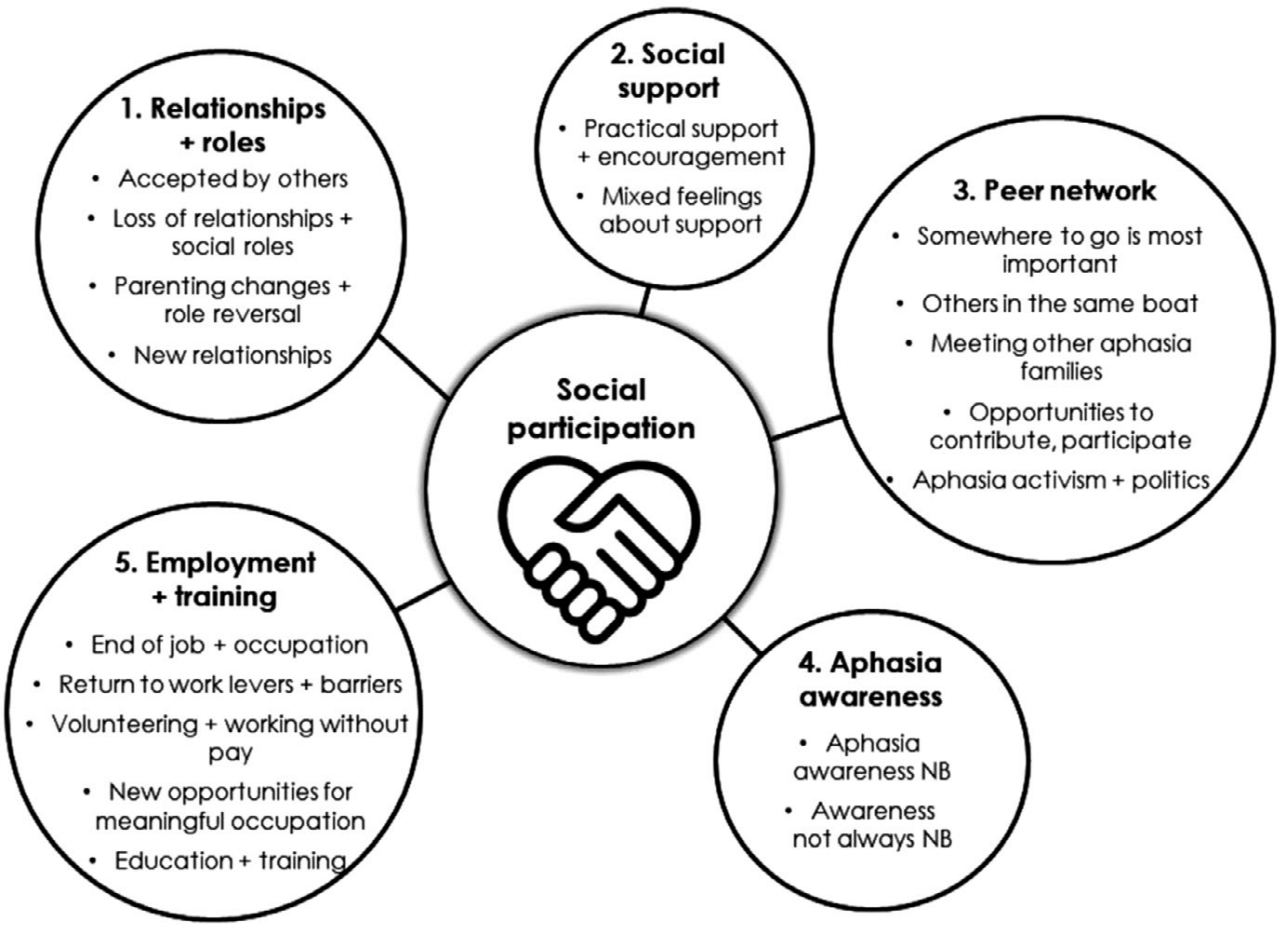
Five themes about social support and participation for LWA

Social participation as relevant to living well with aphasia spanned five themes (Figure 1): Relationships and roles; Social support; Peer network; Aphasia awareness; and Employment and training. Each participant with aphasia was assigned a random identifier from P01‐P14 (corresponding spouses are identified as S01, etc) (see Table 1).

### 3.1 Relationships and roles

This theme describes the importance of relationships and relationship roles in terms of LWA.

#### 3.1.1 Accepted by others

Participants described how family and friends did not treat them differently and were supportive conversation partners, often without formal aphasia information or training.

**S07:** [friend's name] could have a laugh and a craic and a joke and she could understand her from before she had it…she's seeing [P07’s name], the person that was before

**P07:** Yeah

**P10:** They are great about my aphasia … They don't ask about it until I bring it up.

Others were less understanding, as illustrated by P02, who lived alone: ‘I’d say if I said to my sister now ‘do you know what aphasia is?’ she'd say ‘what's that?’ **…**She'd say to me ‘what the hell, talk up’ or something’.

#### 3.1.2 Loss of relationships and social roles

Most PWA experienced relationship changes. P07 experienced role changes in her family and community:

**S07:** She was a leader here in the house… for her family … they used to look up to her and now it's the other way around …

**P07:** Sad.

When P10’s friends withdrew contact, she felt compelled to draw a line under these relationships. **‘**I hated being ghosted … you sent a group text…and you don't get any response’.

Not all PWA had lost friendships as illustrated by P13, ‘I know some people have lost people where I haven't ‐ I've everybody ‐ family and different groups’.

#### 3.1.3 Parenting changes and role reversal

Some participants experienced changes to parenting, role reversal and reduced ability to converse with their children.

**P07:** [S07’s name], ok, yeah but one on one ok but [daughter's name] ok ‐ school ‐ home ‐ homework ‐[S07’s name] always (slightly pointing to S07) – myself (pointing to self) always …

**S07:** …[daughter's name] is confiding in me now.

**P07:** Thank you (annoyed, upset).

**S07:** … Whereas before… she used to go to her mother… she feels she can't ask her mother all that kind of stuff… That's hard.

**P07:** Yeah (crying).

**S07:** And it's a kind of role reversal. Before she used to be telling [daughter's name] what to do and now she's telling her mother what to do. Because her mother doesn't understand sometimes…

**I:** Ok, so there's a lot of change there.

**P07:** Yeah.

Some had to reassert their position as parent and reassure their children:

**P11:** I said (knocks on table) ‘I am the mother here. The boss, the mother… if you don't like it, tough!… I think they were frightened… Like “if I say something my mother will get… worse”…I was always crying’.

#### 3.1.4 New relationships

Many PWA had developed new friendships. Some met new friends through third‐sector support organizations. Not all participants with aphasia were interested in developing new friendships as illustrated by P02 ‘I don't know about “make friends” … I wouldn't be into that’. P10 and P13 were in new romantic relationships. P11 had dated post‐stroke, but her experiences had been largely unsuccessful.

### 3.2 Social support

This theme describes social support and participants’ mixed feelings about support.

#### 3.2.1 Practical support and encouragement

Family and friends were a source of encouragement and practical support (eg with transport, shopping), which helped participation, autonomy and confidence.

**S06:** I taught him the basics… what to do on the bus, where to go … where the shops are…the money part of it.

**P10:** My boyfriend … really did push me because it isn't that he's trying to help me and stuff like that, it's that ‘I can do this’.

#### 3.2.2 Mixed feelings about support

Some PWA expressed frustration at loss of autonomy and dependence on others. P12, a single parent of a young child, felt usurped by her live‐in caregiver:

**I**.**:** And overall, like has it been a positive thing having [Caregiver's name] living with you…Or a mix?..

**P12:** A mix, a mix…

**I:** So …there are good bits, but that you feel a lot more negative about this at the moment?

**P12:** Yes.

**I:** OK. So the negative bits. It's mainly around [Child's name], is that right?

**P12:** [Child's name].

### 3.3 Peer network

This theme describes participant perspectives on being part of a post‐stroke peer network.

#### 3.3.1 Somewhere to go is most important

For many participants, third‐sector support organizations provided ‘a base’, somewhere to go and to meet others and to develop structure and routine, particularly important after job loss. This is illustrated by P06, who valued full‐time employment prior to his stroke, ‘it's somewhere to go is most important… That it kills a few hours in the daytime. And I'm not sitting around the house’.

Participants had variable access to groups and training courses for people with brain injury and/or communication impairment. When available, these included SLT‐led communication groups (either aphasia‐specific or for people with communication impairment more generally), stroke support clubs in the community and third‐sector support organizations for people with brain injury. They provided opportunities to meet others, form a peer network, learn new skills, practice communication, chat about mutual experiences and begin to make sense of and to process feelings. Some, including P04, preferred smaller aphasia/communication focused groups, ‘If we come to the bigger group, it puts us under pressure to contribute’.

The content and programming of groups and courses did not appeal to everyone, including P11 and P02, both of whom lived alone and were not working at the time of interview.

**P11:** I was doing art and wool… it's not my thing.

**P02:** I used to go to that before but I couldn't wear it at all**…** she was talking jargon to me… so I packed it in.

#### 3.3.2 Others in the same boat

Most had opportunities to meet others with aphasia, stroke and/or brain injury through SLTs and third‐sector support organizations. Many described mutual understanding, peer support, relative ease of communication and information‐sharing.

**P04:** There's all sorts of people and they have all sorts of symptoms. But they are in the same boat… there is nothing that can't be talked about… you talk to people that know what you're talking about…

**P06:** We talk about how we all think after we've been sick…if someone talks about something, we all know the same feeling.

PWA did not always bond over shared experiences and meeting others in the same boat was not universally important. P11 described feeling irritated by others attending the third‐sector support organization:

**P11:** they're get on my nerves … I'm not able for it when they're kinda tired they get annoyed ‐ ‘this is my chair!’ (angry voice) ‘this is your chair’ …it's like creche! (laughs).

#### 3.3.3 Meeting other aphasia families

Some highlighted opportunities for family members to meet others for peer support, advice and information: **S01** **‘**it's nice to be mingling with people in the same boat cos they understand.’

#### 3.3.4 Opportunities to contribute, participate

Participants described how SLTs and third‐sector support organizations created opportunities for PWA to contribute and participate in the community including facilitating aphasia groups, communication training, aphasia training for SLT students and healthcare professionals, conversational partner schemes and research participation. This included P08, who had resumed full‐time employment at the time of interview: ‘When I was in Headway, they invited me in to [University name] to speak to a class of speech and language students…I talked away about my experiences. They were able to see where I was struggling to find my words … I really enjoyed it… it's a very easy topic for me to talk about’.

#### 3.3.5 Aphasia activism and politics

Some drew on prior life experience to support and advocate for others. P09 and P13 were politically motivated to improve the nature of stroke and aphasia supports. P09 joked about the possibility of being viewed as a troublemaker having formally complained about his experience of acute care and lobbying for a replacement stroke nurse in his local hospital, ‘they probably have a black mark across my name’.

P09 wanted stroke funding to be separated from cardiovascular health. He described his involvement in organizing an aphasia conference with his local SLT‐led aphasia group. P09 felt that this conference, which had been attended by a government minister, had given a united voice and information to people with more severe aphasia in his aphasia group.

P13 supported local SLTs in facilitating aphasia conversation groups and facilitated discussions between PWA and families as she was able to see both sides. She was also involved in raising aphasia awareness in the community and politically.

**P13:** I never heard the word ‘aphasia’ before this…I just don't think there is anything out there for that…we went to the Dáil (Irish house of government) …And we've gone and talked to the TD's (Teachtaí Dála, Irish parliament members) in way to try and do that. **…** so, there is a little bit but it's not enough…

### 3.4 Aphasia awareness

This theme references perspectives towards the need for increased aphasia awareness in the community and their involvement in awareness‐raising activities.

#### 3.4.1 Aphasia awareness important

Many participants wanted better aphasia awareness in the community, linked with improved communicative access to commercial and public services. Some were involved in delivering communication training to service industry providers.

**P09:** I think there's a program that could be rolled out if it got support… aphasia awareness training ‐ for people who are dealing with the public … that they know what they're dealing with.

#### 3.4.2 Awareness not always important

Many participants, with both mild and severe aphasia, relayed how aphasia did not get in the way of accessing public / commercial services.

**I:** How's the reaction of people like the bus driver or people on the bus?

**P12:** …Em. Chatting.

**I:** And what about in the shops…

**P12:** Good… Very good! …Space.

**I:** going about your business, going into the shops … you don't feel that your aphasia gets in the way?

**P14:** No!

P05 pointed out that you encounter friendly and unfriendly people regardless of aphasia: ‘Some of them are grand, some of them aren't. But then if you meet people like that anyway, some people are great, some are ‐ (laughs)…’.

### 3.5 Employment and training

This theme describes perspectives towards employment issues.

#### 3.5.1 End of job and occupation

Most PWA spoke about employment, which had been negatively affected for most participants. Although 13 participants had been in employment at the time of stroke, only two were working at the time of interview. P08 had returned to his previous position and P10 was now working in a voluntary role. Most participants had experience job losses since having their stroke, including both voluntary and involuntary redundancy and early retirement. Some had attempted unsuccessfully to return to work before deciding to retire. In making this decision, P09 sought advice from others including his manager, GP and Occupational Health. Though ultimately the right decision, it was tinged with sadness for P09 and P13. Job loss often impacted negatively on well‐being, identity, autonomy, financial security and freedom and opportunity to socialize, as illustrated in the excerpts below with participants who had been in full‐time employment prior to stroke.

**P05:** I wouldn't give a damn if I wasn't getting paid either any more at all **…**It was just a craic about it … I used to love going in to that thing… full on…and then you have nothing (gestures to other side).

**P06:** I was always used to that, I was always used to working…I wanted to go back to work after 2 or 3 days.

#### 3.5.2 Return to work—levers and barriers

Perceived barriers in the workplace included a lack of occupational support and equipment and the impact of stress and fatigue. P08 described how he had been facilitated to return to work through the support of his employer, who had maintained open communication throughout his recovery and provided P08 with a flexible, phased return to full‐time working and opportunities to work to his strengths. A lack of flexibility and aphasia awareness reportedly forced P05 out of his much‐loved full‐time vocation. He described what he felt would have helped.

**P05:** Somebody in human resources should have sat down and said …you're good at this, you're good at that… you're not doing that …but it just was you're (unintelligible, pointing to one side) or you're not (pointing to the other). That was it.

**I:** And how do you think that their understanding of aphasia was?

**P05:** They haven't a clue!

#### 3.5.3 Volunteering and working without pay

Several PWA were involved in voluntary activities. P10, who had been employed in a full‐time, high salaried role prior to stroke, found it difficult to gain employment. She described voluntary roles, which did not allow her to use her skillset and provided no opportunity for progression or promotion. This affected her confidence and self‐esteem. In her current managerial role, she was using her skills and training, but was not salaried: **‘**Yeah. I'm able to use all my skills. But I amn't getting paid. That's!’

#### 3.5.4 New opportunities for meaningful occupation

Many participants hoped or planned to return to work in future, including P12, who had not worked since before her stroke.

**P12:** Work. Job… New job. Em. Headway!

**I:** …So are you working towards that in Headway?

**P12:** Yes…

**I:** Why? What's important about that?

**P12:** Head…Positive…Mental. Em. Goals… Drive…Ambitions! …Career!… Money.

Several re‐purposed professional skills to explore new opportunities for employment and/or meaningful and fulfilling occupation (voluntary and remunerated). P13, who took voluntary redundancy, described how using her skillsets to benefit others was stimulating and satisfying. She was less interested in altruism outside her area of expertise.

#### 3.5.5 Education and training

Some participants accessed (or planned) vocational training and further education. P04 linked his instructor's support with his success: ‘He was very encouraging’.

## 4 DISCUSSION

We interviewed working aged PWA, including those with severe aphasia, to explore what supports LWA. This paper focuses on findings relating to social participation and its relevance for LWA. The findings add to a small body of literature examining social participation issues for working aged PWA^28^, ^29^, ^30^ and demonstrate how meaningful, interesting social participation for LWA is necessarily individually defined. Working aged PWA may require flexible support with parenting, accessing a diverse social network and finding opportunities for meaningful social connection, training and employment. Findings are discussed in relation to the existing literature under four headings.

### 4.1 Working aged PWA must be meaningfully involved in setting the agenda for aphasia research, policy and service design

In our study, working aged individuals described diverse experiences of social participation in the context of aphasia and their varied, often opposing preferences and needs around most aspects of social participation including employment, relationships, social support, aphasia awareness and peer networks This resonates with recommendations of Hammel et al that people must ‘be free to define and pursue participation on their own terms rather than meeting predetermined societal norms’^58^ (p.1445).

The findings add to a general lack of description of the actions and perspectives of aphasia activists in the research literature by illustrating the considerable involvement of some working aged participants in ‘grassroots’ community activism and political lobbying. Future research should be conducted in partnership with activists in the aphasia community to explore ideas for a united aphasia voice, potential support needs and to meaningfully include their voice in guiding the aphasia agenda.

### 4.2 Working aged parents with aphasia may have unique support needs

The findings add to a small body of research examining the consequences of aphasia on parenting and children.^12^, ^59^ The data illustrate numerous parenting role changes including changed communication with children, role reversal due to children taking on additional household and caring duties, and some participants having to reassert their authority as parent. These resonate with previous descriptions of problems surfacing many years post‐stroke as children approach adolescence,^12^ changes to the distribution of parenting duties and decisions between PWA and partners,^60^, ^61^ reduced ability to do things with children,^59^ concerns over maintaining custody^63^ and children rejecting the parent with aphasia.^12^ The findings highlight a need for further research examining the impact on parenting with aphasia and the support needs of younger parents with aphasia.

### 4.3 Working aged PWA needs opportunities for paid employment that allows for meaningful contribution and progression

Most participants discussed employment and financial issues, and many had experienced voluntary or involuntary redundancy. Parr previously highlighted how the impact of aphasia on participation is inter‐connected with finances and employment.^12^ We found contrasting experiences of participants who were no longer working. For some, unemployment and reduced income severely restricted social participation; whereas, for others, voluntary redundancy and early retirement did not impact as severely on income and therefore created additional leisure time and opportunity for exploring new, meaningful occupation.

Some valued voluntary employment opportunities to contribute and to re‐purpose and use skills and expertise, particularly when they had lost their previous meaningful, stimulating vocation. Two participants with aphasia were using their extensive skills in mediation and community activism to benefit others, raise awareness and lobby for better support. While the potential negative effects of volunteering have been described previously ^64^ (e.g., fatigue, stress and frustration), our findings elucidate a further issue: lack of remuneration and/or opportunity for progression can impact negatively on the confidence and well‐being of some working aged PWA. There is a need for better support and opportunities for interested PWA to secure employment in paid positions, reflective of their pre‐morbid skills and experiences, for example through the development of appropriate guidelines and joint working between SLTs, voluntary brain injury organizations and return to work agencies.

### 4.4 The aphasia community/peer network does not suit all working aged PWA

The data extend knowledge of the diverse, often opposing experiences and preferences of individual working aged participants. Previous literature has perhaps not emphasized enough the huge differences in the degree to which PWA want to be involved in the aphasia world. Though some working aged PWA greatly enjoyed various aphasia / stroke groups and training courses, these did not interest everybody. This resonates with earlier qualitative studies in which some PWA preferred non‐aphasia specific and/or community‐based interest groups.^61^, ^64^, ^65^ The findings thus support prior recommendations for wider access to general interest, non‐disability specific groups,^67^ but also call for wider access to training courses that better reflect personal and professional interests. This could be achieved, for example, by identifying various evening or adult education courses as aphasia friendly. Further research is necessary to examine how to overcome further education barriers for PWA, including a lack of access to appropriate learning support as previously identified.^68^

Further, previous literature has not highlighted enough the extent to which meaningful social participation for some working aged PWA requires individuals to have communicative access to a wide and flexible network of people. A number of qualitative studies with PWA have described how participation and LWA are supported by skilled and supportive conversation partners,^25^, ^60^, ^61^, ^63^, ^68^, ^69^ and there is evidence that communication partner training (CPT) is effective.^71^ However, CPT, commonly delivered to dyads or small groups, may not be feasible or sufficiently responsive to the needs of young PWA. Some young PWA believe that CPT might not be acceptable to, or appropriate for all family members, and may inadvertently increase peer stigma and rejection.^45^ In the context of working aged PWA, we need to examine how to better support flexible, lifelong social access, acceptance and participation in ‘real‐world’ settings. This is salient considering data highlighting how friendship loss might occur subtly over time through ‘ghosting’ in a WhatsApp group. We need further research examining how to better support younger PWA in navigating a range of different communication formats with friends (e.g. social media and other emerging platforms) including, but not limited to, targeted CPT, supporting PWA to self‐manage communication needs^72^ and social media training.

### 4.5 Methodological critique and limitations

Some potential methodological limitations have been highlighted in an earlier paper.^45^ These include recruitment through service making it less likely to speak with PWA who are not in receipt of social support and potentially at greater risk of reduced social inclusion and participation. Additionally, conducting multiple interviews might have enabled the researcher to review recordings and to identify areas for further expansion at subsequent interviews.^51^ We specifically sought to speak with participants who varied according to time post‐onset to increase the likelihood of hearing the views of people at different stages of recovery. This strategy, however, might limit the reliability of participant reports regarding earlier post‐stroke experiences. Finally, the perspectives of younger PWA (aged 18–30 years) are not included. This has important implications in terms of the representativeness of our findings and a knowledge gap relating to support needs around relationships, education and training, and employment issues for this cohort who may experience unique and long‐lasting impacts on social participation.

### 4.6 Strengths and clinical implications

This study makes a unique contribution by extending our understanding of how best to promote meaningful social participation and personally defined recovery and living well for working aged adults living with post‐stroke aphasia. The findings are generated using a rigorous research design and in collaboration with the PPI aphasia advisory group. Our methodology thus aimed to maximize validity and transferability of the findings, which may have relevance for PWA in other countries, and indeed other patient groups including people with stroke and / or communication impairment from other aetiologies.

The findings underscore how social support and individually defined social participation are critical for working aged adults to live well with aphasia. It is crucial that support for PWA is extended to the support networks of PWA, and that social participation outcomes at all stages of recovery are explicitly targeted as part of health policy and stroke and SLT practice. Such support must be designed and evaluated in collaboration with working aged PWA to better meet the diverse and unique needs of a younger population in the context of living a longer number of years with stroke. Support needs may include, for example, support with parenting, employment, training and education in the context of aphasia, and self‐management support to access a wide and diverse social network, including via digital technology.

## 5 CONCLUSION

We interviewed working aged PWA, including those with severe aphasia, to explore what has or would help them to live well. This paper focuses on the relevance of social support and participation for working aged individuals to LWA. Meaningful participation was individually defined. Working aged PWA must be meaningfully involved at all stages from the outset of intervention development. Participation was promoted by opportunities for social connection, employment, contribution and progression. Working aged PWA need support to secure employment that offers opportunity for meaningful contribution, progression and remuneration. Finally, working aged adults have a wide, diverse social network and we need to better understand how to support social participation in this context, including via individualized self‐management support.

## CONFLICT OF INTEREST

The authors declare no competing interests.

## AUTHOR CONTRIBUTIONS

MM, AM, AH and SF involved in conceptualization and design. MM performed the acquisition of data and wrote the manuscript. MM, AM, AH and SF performed the methodology. MM and SF analysed and interpreted the data, and involved in project administration. MM, AM, AH, SF and RG critically wrote, reviewed and edited the manuscript.

## Supporting information

Supplementary_file_1

Supplementary_file_2

Supplementary_file_3

## Data Availability

This qualitative interview study generated rich, personal information about participants. It is not possible to make these data available in such a way that would protect the anonymity of participants required for research ethical approval.

## References

[1] Kagan A, Simmons‐Mackie N. From my perspective: changing the aphasia narrative. ASHA Leader. 2013;18(11):6‐8. 10.1044/leader.FMP.18112013.6

[2] Aphasia Institute . What is aphasia? Masked competence ‐ knowing more than you can say 2015 [01.06.2020]. Available from: https://www.aphasia.ca/home‐page/about‐aphasia/what‐is‐aphasia/. Accessed June 1, 2021.

[3] Baker C, Worrall L, Rose M, Ryan B. ‘It was really dark’: the experiences and preferences of people with aphasia to manage mood changes and depression. Aphasiology. 2020;34(1):19‐46. 10.1080/02687038.2019.1673304

[4] Grawburg M, Howe T, Worrall L, Scarinci N. Third‐party disability in family members of people with aphasia: a systematic review. Disabil Rehabil. 2013;35(16):1324‐1341. 10.3109/09638288.2012.735341 23826903

[5] Black‐Schaffer R, Osberg J. Return to work after stroke: development of a predictive model. Arch Phys Med Rehabil. 1990;71:285‐290. 2327878

[6] Hilari K, Needle J, Harrison K. What are the important factors in health‐related quality of life for people with aphasia? A systematic review. Arch Phys Med Rehabil. 2012;93(1 SUPP):S86‐S95. 22119074 10.1016/j.apmr.2011.05.028

[7] Howe T, Davidson B, Worrall L, et al. 'You needed to rehab … families as well': family members' own goals for aphasia rehabilitation. Int J Lang Commun Disord. 2012;47(5):511‐521. 10.1111/j.1460-6984.2012.00159.x 22938062

[8] Kelly H, Kennedy F, Britton H, McGuire G, Law J. Narrowing the “digital divide”—facilitating access to computer technology to enhance the lives of those with aphasia: a feasibility study. Aphasiology. 2016;30(2–3):133‐163. 10.1080/02687038.2015.1077926

[9] Morris J, Franklin S, Menger F. Returning to work with aphasia: A case study. Aphasiology. 2011;25(8):890‐907. 10.1080/02687038.2010.549568

[10] Northcott S, Marshall J, Hilari K. What factors predict who will have a strong social network following a stroke? J Speech Lang Hear Res. 2016;59(4):772‐783. 10.1044/2016_JSLHR-L-15-0201 27401538

[11] Northcott S, Moss B, Harrison K, Hilari K. A systematic review of the impact of stroke on social support and social networks: associated factors and patterns of change. Clin Rehabil. 2016;30(8):811‐831. 10.1177/0269215515602136 26330297

[12] Parr S. Psychosocial aspects of aphasia: Whose perspectives? Folia Phoniatrica Et Logopaedica. 2001;53(5):266‐288. 10.1159/000052681 11464068

[13] Parr S. Coping with aphasia: Conversations with 20 aphasic people. Aphasiology. 1994;8(5):457‐466. 10.1080/02687039408248670

[14] Parr S. Living with severe aphasia: Tracking social exclusion. Aphasiology. 2007;21(1):98‐123. 10.1080/02687030600798337

[15] Shadden B. Aphasia as identity theft: Theory and practice. Aphasiology. 2005;19(3–5):211‐223. 10.1080/02687930444000697

[16] Carragher M, Steel G, O’Halloran R, et al. Aphasia disrupts usual care: the stroke team’s perceptions of delivering healthcare to patients with aphasia. Disabil Rehabil. 2020;1–12. 10.1080/09638288.2020.1722264 32045533

[17] Kagan A, Simmons‐Mackie N, Rowland A, et al. Counting what counts: A framework for capturing real‐life outcomes of aphasia intervention. Aphasiology. 2008;22(3):258‐280. 10.1080/02687030701282595

[18] Chapey R, Duchan J, Elman R, et al. Life participation approach to aphasia: A statement of values for the future. In: Chapey R, ed. Language Intervention Strategies in Aphasia and Related Neurogenic Communication Disorders. Philadelphia: Lippincott Williams & Wilkins; 2001:235‐245.

[19] Howe T. Found opportunities for social participation: facilitating inclusion of adults with aphasia. Topics in Language Disorders. 2017;37(1):38–51.

[20] Cruice M, Worrall L, Hickson L. Quantifying aphasic people's social lives in the context of non‐aphasic peers. Aphasiology. 2006;20(12):1210‐1225. 10.1080/02687030600790136

[21] Byng S, Duchan JF. Social model philosophies and principles: Their applications to therapies for aphasia. Aphasiology. 2005;19(10–11):906‐922. 10.1080/02687030544000128

[22] Simmons‐Mackie N, Damico J. Access and social inclusion in aphasia: interactional principles and applications. Aphasiology. 2007;21(1):81‐97. 10.1080/02687030600798311.

[23] Kagan A. Supported conversation for adults with aphasia: methods and resources for training conversation partners. Aphasiology. 1998;12(9):816‐830. 10.1080/02687039808249575

[24] Manning M, MacFarlane A, Hickey A, Franklin S. Perspectives of people with aphasia post‐stroke towards personal recovery and living successfully: a systematic review and thematic synthesis. PLoS One. 2019;14(3):e0214200. 10.1371/journal.pone.0214200 30901359 PMC6430359

[25] Mc Menamin R, Tierney E, Mac FA. Addressing the long‐term impacts of aphasia: how far does the Conversation Partner Programme go? Aphasiology. 2015;29(8):889‐913. 10.1080/02687038.2015.1004155

[26] McMenamin R, Tierney E, MacFarlane A. Using a participatory learning and action (PLA) research approach to involve people with aphasia as co‐researchers in service evaluation: an analysis of co‐researchers’ experiences. Aphasiology. 2018;32(sup1):142‐144. 10.1080/02687038.2018.1486380

[27] Mc Menamin R, Tierney E, Mac FA. Who decides what criteria are important to consider in exploring the outcomes of conversation approaches? A participatory health research study. Aphasiology. 2015;29(8):914‐938. 10.1080/02687038.2015.1006564

[28] Pound C. An Exploration of the Friendship Experiences of Working‐Age Adults with Aphasia [PhD]. London, UK: Brunel University; 2013.

[29] Graham JR, Pereira S, Teasell R. Aphasia and return to work in younger stroke survivors. Aphasiology. 2011;25(8):952‐960. 10.1080/02687038.2011.563861

[30] Dalemans RJP, De Witte LP, Wade DT, Van den Heuvel WJA. A description of social participation in working‐age persons with aphasia: a review of the literature. Aphasiology. 2008;22(10):1071‐1091. 10.1080/02687030701632179

[31] McElwaine P, McCormack J, Harbison J. On behalf of the National Stroke Programme Audit Steering Group IHF/HSE National Stroke Audit 2015. Dublin: Irish Heart Foundation (IHF) & Health Service Executive; 2015.

[32] Health Service Executive . Planning for Health: Trends and Priorities to Inform Health Service Planning 2016. Dublin: Health Service Executive; 2016.

[33] Béjot Y, Delpont B, Giroud M. Rising stroke incidence in young adults: more epidemiological evidence, more questions to be answered. J Am Heart Assoc. 2016;5(5):e003661. 10.1161/JAHA.116.003661 27169549 PMC4889213

[34] Palmer R, Witts H, Chater T. What speech and language therapy do community dwelling stroke survivors with aphasia receive in the UK? PLoS One. 2018;13(7):e0200096. 10.1371/journal.pone.0200096 29990345 PMC6039008

[35] Rose M, Ferguson A, Power E, Togher L, Worrall L. Aphasia rehabilitation in Australia: current practices, challenges and future directions. Int J Speech Lang Pathol. 2014;16(2):169‐180. 23777446 10.3109/17549507.2013.794474

[36] Verna A, Davidson B, Rose T. Speech‐language pathology services for people with aphasia: a survey of current practice in Australia. Int J Speech Lang Pathol. 2009;11(3):191‐205.

[37] Manning M, Cuskelly C, Russ E, Franklin S. Supporting people with post‐stroke aphasia to live well: a cross‐sectional survey of Speech & Language Therapists in Ireland. Health Soc Care Community. 2020;28(6):2117‐2124. 10.1111/hsc.13021 32462685

[38] Horgan F, Walsh M, Galvin R, Macey C, Loughnane C. National Survey of Stroke Survivors 2013: Experiences and long‐term needs reported by stroke survivors living in the community in Ireland. Dublin: National Disability Authority/Irish Heart Foundation; 2014.

[39] Code C, Heron C. Services for aphasia, other acquired adult neurogenic communication and swallowing disorders in the United Kingdom, 2000. Disabil Rehabil. 2003;25(21):1231‐1237. 10.1080/09638280310001599961 14578063

[40] Katz RC, Hallowell B, Code C, et al. A multinational comparison of aphasia management practices. Int J Lang Commun Disord. 2000;35(2):303‐314. 10.1080/136828200247205 10912257

[41] Kong A, Tse C. Clinician survey on speech pathology services for people with aphasia in hong kong. Clin Arch Commun Disord. 2018;3(3):201‐212.

[42] Kong A. Family members' report on speech‐language pathology and community services for persons with aphasia in Hong Kong. Disabil Rehabil. 2011;33(25–26):2633‐2645. 10.3109/09638288.2011.579220 22082073

[43] Community Services Subgroup of the National Stroke Working Group . Community Stroke Services Survey Report 4 Summary Report. Dublin: HSE Strategy and Programmes Directorate; 2011.

[44] Clark A, Lissel S, Davis C. Complex critical realism: Tenets and application in nursing research. Adv Nurs Sci. 2008;31:E67‐79. 10.1097/01.ANS.0000341421.34457.2a 19033741

[45] Manning M, Mac Farlane A, Hickey A, Galvin R, Franklin S. The relevance of stroke care for living well with post‐stroke aphasia: qualitative interviews with working‐aged adults. Disabil Rehabil. 2020. 10.1080/09638288.2020.1863483 33356970

[46] O'Brien BC, Harris IB, Beckman TJ, Reed DA. Standards for reporting qualitative research: a synthesis of recommendations. Acad Med. 2014;89(9):1245‐1251. 24979285 10.1097/ACM.0000000000000388

[47] Creswell JW. Research Design: Qualitative, Quantitative, and Mixed Methods Approaches, 3rd edn. London: Sage; 2009.

[48] Goodglass H, Kaplan E, Barresi B. Boston Diagnostic Aphasia Examination, 3rd edn. San Antonio, TX: Pearson; 2000.

[49] Kertesz A. Western Aphasia Battery‐Revised (WAB‐R). San Antonio, TX: Pearson; 2006.

[50] Swinburn K, Porter G, Howard D. Comprehensive Aphasia Test. Oxford: Psychology Press; 2005.

[51] Carlsson E, Paterson BL, Scott‐Findlay S, Ehnfors M, Ehrenberg A. Methodological issues in interviews involving people with communication impairments after acquired brain damage. Qual Health Res. 2007;17:1361‐1371. 18000075 10.1177/1049732307306926

[52] Dalemans R, Wade DT, van den Heuvel WJ , de Witte LP . Facilitating the participation of people with aphasia in research: a description of strategies. Clin Rehabil. 2009;23:948‐959. 19570814 10.1177/0269215509337197

[53] Berg K, Askim T, Balandin S, Armstrong E, Rise MB. Experiences of participation in goal setting for people with stroke‐induced aphasia in Norway. A qualitative study. Disabil Rehabil. 2017;39(11):1122‐1130. 10.1080/09638288.2016.1185167 27293106

[54] Braun V, Clarke V. Using thematic analysis in psychology. Qual Res Psychol. 2006;3(2):77‐101. 10.1191/1478088706qp063oa

[55] Clarke V, Braun V. Thematic Analysis. In: Lyons E, Coyle A, eds. Analysing Qualitativa Data in Psychology, 2nd edn. London: SAGE; 2015:84‐103.

[56] Braun V, Clarke V. Reflecting on reflexive thematic analysis. Qual Res Sport Exerc Health. 2019;11(4):589‐597. 10.1080/2159676X.2019.1628806

[57] Terry G, Doing thematic analysis. In: Lyons E, Coyle A, eds. Analysing Qualitative Data in Psychology, 2nd edn. London: SAGE; 2015:104‐118.

[58] Hammel J, Magasi S, Heinemann A, Whiteneck G, Bogner J, Rodriguez E. What does participation mean? An insider perspective from people with disabilities. Disabil Rehabil. 2008;30(19):1445‐1460. 10.1080/09638280701625534 18923977

[59] Fotiadou D, Northcott S, Chatzidaki A, Hilari K. Aphasia blog talk: How does stroke and aphasia affect a person’s social relationships? Aphasiology. 2014;28(11):1281‐1300. 10.1080/02687038.2014.928664

[60] Le Dorze G, Salois‐Bellerose É, Alepins M, Croteau C, Hallé M‐C. A description of the personal and environmental determinants of participation several years post‐stroke according to the views of people who have aphasia. Aphasiology. 2014;28(4):421‐439. 10.1080/02687038.2013.869305

[61] Dalemans RJ, de Witte L , Wade D, van den Heuvel W . Social participation through the eyes of people with aphasia. Int J Lang Commun Disord. 2010;45(5):537‐550. 10.3109/13682820903223633 19839875

[62] Brown K, Worrall L, Davidson B, Howe T. Snapshots of success: An insider perspective on living successfully with aphasia. Aphasiology. 2010;24(10):1267‐1295. 10.1080/02687031003755429

[63] Pearl G, Sage K, Young A. Involvement in volunteering: an exploration of the personal experience of people with aphasia. Disabil Rehabil. 2011;33(19–20):1805‐1821. 10.3109/09638288.2010.549285 21859420

[64] Bright FAS, Kayes NM, McCann CM, McPherson KM. Hope in people with aphasia. Aphasiology. 2013;27(1):41‐58. 10.1080/02687038.2012.718069

[65] Armstrong E, Hersh D, Hayward C, Fraser J. Communication disorders after stroke in Aboriginal Australians. Disabil Rehabil. 2015;37(16–17):1462‐1469. 10.3109/09638288.2014.972581 25365701

[66] Rotherham A, Howe T, Tillard G. “We just thought that this was Christmas”: perceived benefits of participating in aphasia, stroke, and other groups. Aphasiology. 2015;29(8):965‐982. 10.1080/02687038.2015.1016887

[67] Bruce C, Parker A, Renfrew L. ‘Helping or something’: perceptions of students with aphasia and tutors in further education. Int J Lang Commun Disord. 2006;41(2):137‐154. 10.1080/13682820500224125 16546892

[68] Howe TJ, Worrall LE, Hickson LMH. Interviews with people with aphasia: Environmental factors that influence their community participation. Aphasiology. 2008;22(10):1092‐1120. 10.1080/02687030701640941

[69] Niemi T, Johansson U. The lived experience of engaging in everyday occupations in persons with mild to moderate aphasia. Disabil Rehabil. 2013;35(21):1828‐1834. 10.3109/09638288.2012.759628 23350760

[70] Simmons‐Mackie N, Raymer A, Cherney LR. Communication partner training in aphasia: an updated systematic review. Arch Phys Med Rehabil. 2016;97(12):2202‐2221.e8. 10.1016/j.apmr.2016.03.023 27117383

[71] Wray F, Clarke D. Longer‐term needs of stroke survivors with communication difficulties living in the community: a systematic review and thematic synthesis of qualitative studies. BMJ Open. 2017;7(10). 10.1136/bmjopen-2017-017944 PMC5640038 28988185

