# Supplementary material for: ‘I hated being ghosted’ – The relevance of social participation for living well with post‐stroke aphasia: Qualitative interviews with working aged adults
Source: Health Expect. 2021 Jun 15;24(4):1504–15. doi: 10.1111/hex.13291 (PMC8369109; doi:10.1111/hex.13291)
Supplement: Supplementary file 1 — Supplementary_file_1 [file HEX-24-1504-s003.docx]

# Appendix 1 Standards for reporting qualitative research (SRQR)

From: O'Brien BC, Harris IB, Beckman TJ, Reed DA. Standards for reporting qualitative research: A synthesis of recommendations. Acad Med. 2014;89(9):1245-51.

| **Item category** | **Checklist item** | **Explanation** | **How / where item is addressed** |
| --- | --- | --- | --- |
| **Title and abstract** | S1Title | Concise descrdiption of the nature and topic of the study identifying the study as qualitative or indicating th eapproach (e.g. ethnography, grounded theory) or data collection methods is recommended. | … the relevance of social participation for living well with post-stroke aphasia: Qualitative interviews with working-aged adults. |
|  | S2 Abstract | Summary of key elements of the study using the abstract format of intended publication. | Item is addressed – see Abstract |
| **Introduction** | S3 Problem formulation | Description and significance of the problem/phenomenon studied; review of relevant theory and empirical work; problem statement. | See Introduction section. |
|  | S4 Purpose or research question | Purpose of the study and specific objectives or questions. | See end of introduction. |
| **Methods** | S5 Qualitative approach and research paradigm | Qualitative approach and guiding theory if appropriate; identifying the resaerch paradigm is also recommended; rationale. | See Qualitative approach and research paradigm section. |
|  | S6 Researcher characteristics and reflexivity | Researchers’ characteristics that may influence the resaerch including personal attributes, qualifications/experience, relationship with participants, assumptions and/or presuppositions, potential or actual interaction between researchers’ characteristics and the research questions, approach, methods, results, and/or transferability. | See Research team characteristics and reflexivity section. |
|  | S7 Context | Setting/site and salient contextual factors; rationale. | See sampling strategy for gatekeepers. |
|  | S8 Sampling strategy | How and why research participants, documents, or events were slected; criteria for deciding when no further sampling was necessary; rationale. | See Sampling strategy section. |
|  | S9 Ethical issues pertaining to human subjects | Documentation of approval by an appropriate ethics review board and participant consent, or explanation for lack thereof; other confidentiality and data security issues. | See Ethical issues section and Appendix 2 Recruitment pack. |
|  | S10 Data collection methods | Types of data collected; details of data collection procedures including (as appropriate) start and stop dates of data collection and analysis, iterative process, triangulation of sources/methods, and modificatino of procedures in resonse to evolving study findings; rationale. | See Data collection methods section. |
|  | S11 Data collection instruments and technologies | Description of instruments (e.g. interview guides, questionnaires) and devices used for data collection. | See Data collection methods section and Appendix 3 Topic guide. |
|  | S12 Units of study | Number and relevant characteristics of participants, documetns or events included in the study; level of participation (could be reported in results). | See Participants section. |
|  | S13 Data processing | Methods for processing data prior to and during analysis, including transcrition, data entry, data management and security, verification of data integrity, data coding and anonymisation/deidentification of excerpts. | See Data processing and analysis section. |
|  | S14 Data analysis | Process by which inferences, themes were identified and developed, including the researchers involved in data analysis; usually references a specific paraidm or approach; rationale. | See Data processing and analysis section and Appendices 5 + 6. |
|  | S15 Techniques to enhance trustworthiness | Techniques to enhance trustworthiness and credibility of data analysis (e.g. member checking, audit trail, triangulation); rationale. | See Techniques to enhance trustworthiness section. |
| **Results / findings** | Synthesis and interpretation | Main findings | See Results section + figures. |
|  | Links to empirical data | Evidence to substantiate analytic findings | See participant quotes throughout Results section. |
| **Discussion** | S18 | Integration with prior work, implications, transfereability, and contribution(s) to the field. | See Discussion section. |
|  | S19 | Trustworthiness and limitations of findings | See Methodological critique and limitations section. |
| **Other** | S20 Conflicts of interest | Potential sources of influence or perceived influence on study conduct and conclusions; how these were managed. | See Conflicts of interest statement. |
|  | S21 Funding | Sources of funding and other support; role of funders in data collection, interpretation and reporting. | See Funding statement |
