# Supplementary material for: ‘I hated being ghosted’ – The relevance of social participation for living well with post‐stroke aphasia: Qualitative interviews with working aged adults
Source: Health Expect. 2021 Jun 15;24(4):1504–15. doi: 10.1111/hex.13291 (PMC8369109; doi:10.1111/hex.13291)
Supplement: Supplementary file 2 — Supplementary_file_2 [file HEX-24-1504-s001.docx]

# Appendix 2 Aphasia-friendly recruitment pack

**STUDY INFORMATION**

**Study title**

Who or what helps people with aphasia post-stroke of working age to live well in Ireland?

**Research team**

PhD Scholar, Molly Manning (University of Limerick)

**Supervised by:**

Professor Sue Franklin (University of Limerick)

Professor Anne Hickey (Royal College of Surgeons in Ireland)

Professor Anne MacFarlane (University of Limerick)

**What is the study about?**

To find out about who or what helps people with aphasia post-stroke of working age to live well in Ireland.

If you agree, your significant other (e.g. spouse, partner, son/daughter or main caregiver) will also be included.

**Where will the study take place?**

In your home or in [Gatekeeper facility] at a time that suits you.

**What will I have to do?**

The study has 2 parts. You will be seen by PhD student Molly Manning on 2 different days. There will be at least 2 days between sessions.

On the first day

- You will do a small number of tests, such as naming pictures of objects or describing more complex pictures. This will take about 1 hour.
- We will audio-record and video-record your picture naming - these recordings will be destroyed once the recording has been transcribed.

On the second day

- You will be asked about your experiences and opinions.
- If you agree, your significant other will also answer questions.
- You will both be interviewed in the same room at the same time.
- You will be asked questions separately.
- The interview will be audio-recorded and video-recorded.
- The interview will take 2 hours, with breaks if you need them.

**Who can be in the study?**

- People who have aphasia after a stroke:
  - Aged 18-65 years.
  - English-speakers.
  - No severe hearing or cognitive problems.
  - It has been at least 1 year since the stroke.
- If you agree, a significant other such as your spouse, partner, son/ daughter or main caregiver.

**Do I have to do this research?**

No. And it will not affect access to any future treatment.

**Can I change my mind?**

YES – AT ANY TIME

**What are the benefits of taking part in the study?**

There is no pay.

The study will help us to understand who or what has been important for you to live well. This is important for planning support services for people with aphasia.

**Are there any risks?**

There are no risks. If you are tired or emotional during the study, you can take a break or stop whenever you want

**Is the research confidential?**

- YES - Your information will be kept private.
- The information you give will be used as part of a larger study.
- The results may be presented at a conference and in a scientific journal.
- The things that you say may also be presented.
- You will never be named in the research.
- The recordings will not be presented.
- Information will be destroyed after 7 years.
- You can ask for a copy of the audio-recording, video-recording, transcript of the interview and the research findings.
- You can review these to make sure that we have heard you correctly.

**Who do I contact if I am interested in taking part? [GATEKEEPER WILL DECIDE IF POTENTIAL PARTICIPANTS CONTACT GATEKEEPER OR RESEARCHER DIRECTLY]**


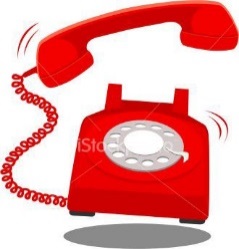
Molly Manning (PhD Student)

**Phone:** 061-202113


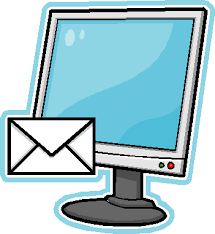


**Email:** [molly.manning@ul.ie](mailto:molly.manning@ul.ie)


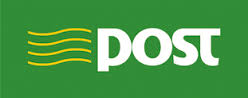


**Address:** School of Allied Health,

Faculty of Education and Health Sciences,

University of Limerick.

**Can I complain?**

YES

Complaints or queries about the study can be directed to:

Professor Sue Franklin,

Professor of Speech & Language Therapy,

School of Allied Health,

University of Limerick.

OR

Chairman,

Education and Health Sciences Research Ethics Committee

EHS Faculty Office

University of Limerick

Tel: (061) 234 101

Email: [ehsresearchethics@ul.ie](mailto:ehsresearchethics@ul.ie)

**This research has received ethical approval from the University of Limerick Research Ethics Committee.**

EHSREC approval number: 2016_09_06_EHS

**CONSENT FORM**

**Study title: Who or what helps people with aphasia post-stroke of working age to live well in Ireland?**

| I **read** the **information leaflet** | YES [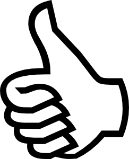](javascript:edit(29226)) | NO 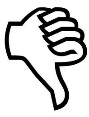 |
| --- | --- | --- |
| I had an **opportunity** to ask **questions** | YES [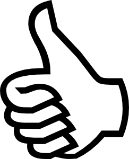](javascript:edit(29226)) | NO 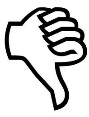 |
| I was **satisfied** with the **answers** to **my questions** | YES [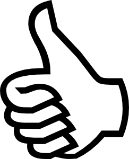](javascript:edit(29226)) | NO 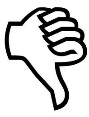 |
| I understand what is **involved** | YES [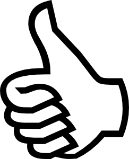](javascript:edit(29226)) | NO 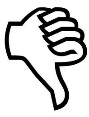 |
| I understand that **information** is kept **safe** and private | YES [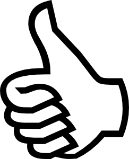](javascript:edit(29226)) | NO 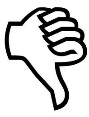 |
| I know that I **will not be named** in any **reports** or **talks** about this study | YES [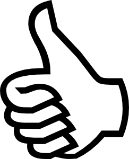](javascript:edit(29226)) | NO 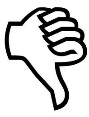 |
| I know I can stop at any time | YES [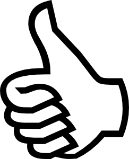](javascript:edit(29226)) | NO 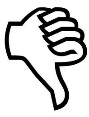 |
| I agree to my spouse or carer being interviewed | YES [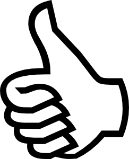](javascript:edit(29226)) | NO 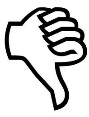 |
| I agree to being audio-recorded | YES [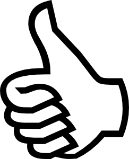](javascript:edit(29226)) | NO 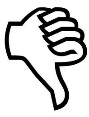 |
| I agree to being video-recorded | YES [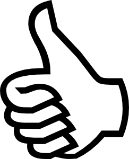](javascript:edit(29226)) | NO 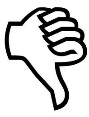 |
| I understand that I can ask for a copy of the **audio-recording**, the **video-recording**, the **interview transcript** and the **research findings**.  I can **review** these to make sure that I have been **heard correctly**. | YES [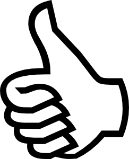](javascript:edit(29226)) | NO 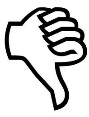 |

**I agree** to participate in this study

Name: (please print): __________________________

___________________________________ ________________________

You Sign Here Date

___________________________________ ________________________

Researcher Signs Here Date

**Contacts**


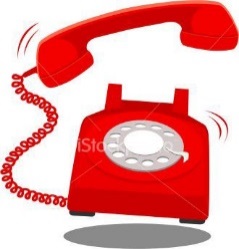
Molly Manning (PhD Student)

**Phone:** 061-202113


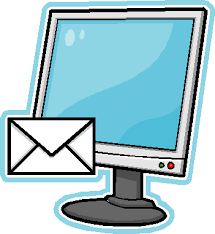


**Email:** [molly.manning@ul.ie](mailto:molly.manning@ul.ie)


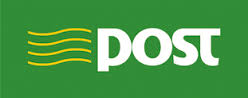


**Address:** School of Allied Health,

Faculty of Education and Health Sciences,

University of Limerick.

**This research has received ethical approval from the University of Limerick Research Ethics Committee.**

EHSREC approval number: 2016_09_06_EHS.

**If you have concerns regarding this study, please contact:**

Chairperson,

Education and Health Sciences Research Ethics Committee

EHS Faculty Office

University of Limerick

Tel: (061) 234 101

Email: [ehsresearchethics@ul.ie](mailto:ehsresearchethics@ul.ie)
