# Supplementary material for: ‘I hated being ghosted’ – The relevance of social participation for living well with post‐stroke aphasia: Qualitative interviews with working aged adults
Source: Health Expect. 2021 Jun 15;24(4):1504–15. doi: 10.1111/hex.13291 (PMC8369109; doi:10.1111/hex.13291)
Supplement: Supplementary file 3 — Supplementary_file_3 [file HEX-24-1504-s002.pdf]

Interview study

findings

1

- I interviewed **14 people with aphasia**.
  - **8 men** and **6 women**
  - 4 spouses
  - **In Galway, Limerick, Clare, South Tipperary, Dublin**
  - From **Headway, ABI Ireland, Irish Heart Foundation, Croí, Aphasia Ireland, SLT's**

2

- They had a stroke **14 months** - **14 years ago.**
- They are **aged 32 – 62 years.**
- **6** have **severe** aphasia.
- **5** have **moderate** aphasia.
- **3** have **mild** aphasia.

3

Interview study findings

- ✓ I will now **present** the **findings**.
- ✓ Then **you can** give your **comments** and **opinions.**
- ✓ **Ideas** will be written down on **post-its** as a record of what everybody says.

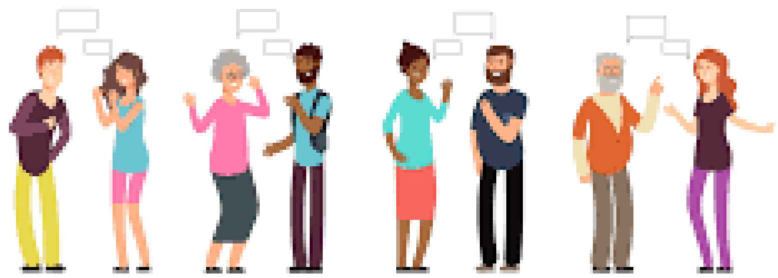

4

Interview study findings

Stroke Care:

- People had **different experiences** of care, **good + bad**.
- Some had **no support after hospital**, others had **lots**.
- Many people valued:
  - **Counselling** or emotional support
  - **Speech Therapy**
  - **Healthcare staff** to **know about apha**sia
  - **Information** about **services**.

5

✓ Do you have **any comments** or **questions**?

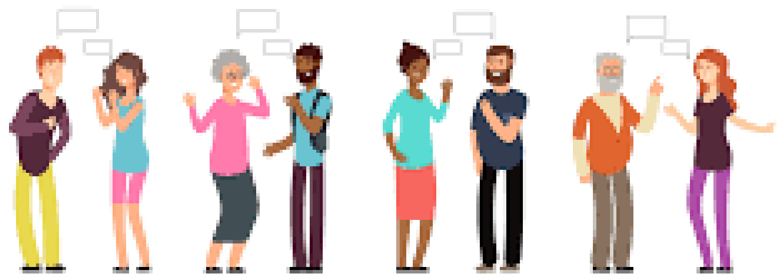

6

Interview study findings

Aphasia information

- Most people valued:
  - **Aphasia information** – even years after stroke.
  - **Information** and **training for families, caregivers.**

7

✓ Do you have **any comments** or **questions**?

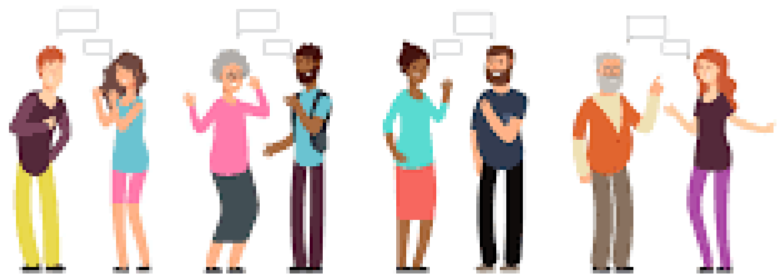

8

Interview study findings

Support for families

- **Many spouses** experienced **loss and life changes**.
- Many people valued:
  - **Counselling** or emotional support for **spouses, kids**.
  - **Stroke Clubs** and **voluntary organisations** to include **families**.

9

✓ Do you have **any comments** or **questions**?

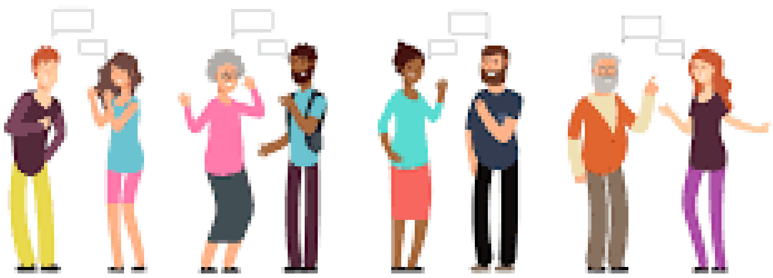

10

Interview study findings

Practical support

- Many people valued:
  - Having a **Key Worker** (e.g. in Headway, ABI).
  - **Social support** from family and friends.
  - **Support with housework** from caregivers or home help.
  - People had **mixed feelings** about **social support**.

11

✓ Do you have **any comments** or **questions**?

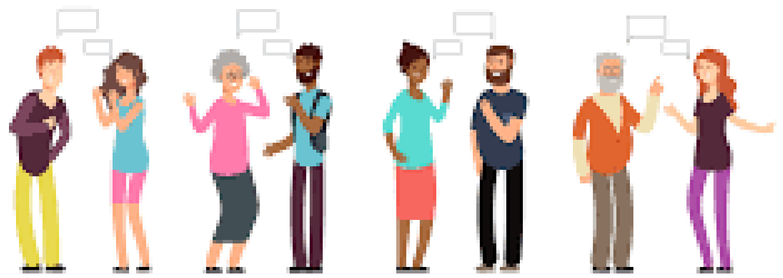

12

Interview study findings

Moving on

- Some became **more comfortable** and **confident** with aphasia.
- Many people valued:
  - **Getting out** and **meeting people**.
  - Having **somewhere to go**.
  - Using **communication strategies**.
  - Having a **positive outlook**.

13

✓ Do you have **any comments** or **questions**?

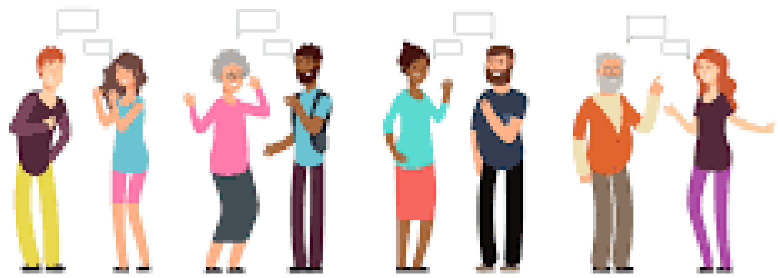

14

Interview study findings

Returning to Work / Education

- Many people **no longer worked**.
- For some, this meant **less money** + **less socialising**.
- Some people had **returned to work**.
- Others **wanted to** in the future.
- People had **good + bad experiences** of returning to work.
- Many valued **further education** and **training courses**.

15

✓ Do you have **any comments** or **questions**?

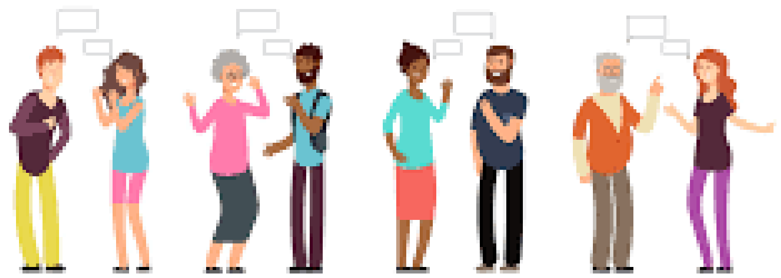

16

Interview study findings

Aphasia Community

- Meeting **others with aphasia** was important:
  - **Shared experience**
  - **Advice** + information
- Some people were involved in:
  - **Raising awareness** of aphasia + **political lobbying**
  - **Aphasia training**
  - **Facilitating aphasia groups**

17

✓ Do you have **any comments** or **questions**?

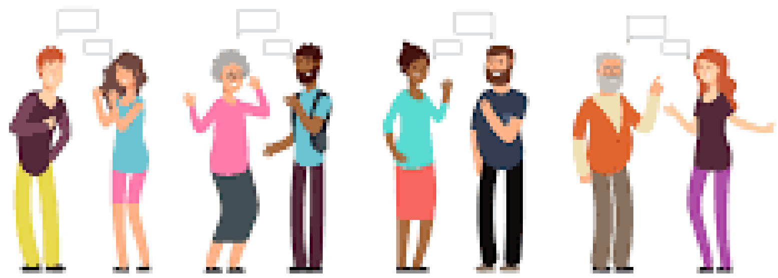

18

Interview study findings

People need different things at different times

- ✓ Stroke care
- ✓ Aphasia information
- ✓ Support for families
- ✓ Practical support
- ✓ Moving on
- ✓ Returning to work / Education
- ✓ Aphasia community

Is there anything missing?

19
